# Expression profiling of sexually dimorphic genes in the Japanese quail, *Coturnix japonica*

**DOI:** 10.1038/s41598-020-77094-y

**Published:** 2020-11-30

**Authors:** Miki Okuno, Shuntaro Miyamoto, Takehiko Itoh, Masahide Seki, Yutaka Suzuki, Shusei Mizushima, Asato Kuroiwa

**Affiliations:** 1 School of Life Science and Technology, Tokyo Institute of Technology, 2-12-1 Ookayama, Meguro-ku, Tokyo, 152-8550 Japan; 2 Biosystems Science Course, Graduate School of Life Science, Hokkaido University, Kita 10, Nishi 8, Kita-ku, Sapporo, Hokkaido 060-0810 Japan; 3 Department of Computational Biology and Medical Sciences, The University of Tokyo, 5-1-5 Kashiwanoha, Kashiwa, 277-8562 Japan; 4 Division of Reproductive and Developmental Biology, Department of Biological Sciences, Faculty of Science, Hokkaido University, Kita 10 Nishi 8, Kita-ku, Sapporo, Hokkaido 060-0810 Japan

**Keywords:** Development, Gene expression

## Abstract

Research on avian sex determination has focused on the chicken. In this study, we established the utility of another widely used animal model, the Japanese quail (*Coturnix japonica*), for clarifying the molecular mechanisms underlying gonadal sex differentiation. In particular, we performed comprehensive gene expression profiling of embryonic gonads at three stages (HH27, HH31 and HH38) by mRNA-seq. We classified the expression patterns of 4,815 genes into nine clusters according to the extent of change between stages. Cluster 2 (characterized by an initial increase and steady levels thereafter), including 495 and 310 genes expressed in males and females, respectively, contained five key genes involved in gonadal sex differentiation. A GO analysis showed that genes in this cluster are related to developmental processes including reproductive structure development and developmental processes involved in reproduction were significant, suggesting that expression profiling is an effective approach to identify novel candidate genes. Based on RNA-seq data and in situ hybridization, the expression patterns and localization of most key genes for gonadal sex differentiation corresponded well to those of the chicken. Our results support the effectiveness of the Japanese quail as a model for studies gonadal sex differentiation in birds.

## Introduction

In birds, males are homogametic (ZZ) and females are heterogametic (ZW). The sex determination mechanism involving gene(s) on the sex chromosome is highly conserved among birds. In chickens, sex determination is thought to occur after embryonic day (E) 4.5, corresponding to Hamburger and Hamilton stage^1^ 24 (HH24). After sex determination, the gonads differentiate to testes or ovaries according to the sex chromosomal constitution of cells. However, until around E6–6.5 (HH29), the gonads are considered “bipotential,” indicating that they are able to differentiate into either testes or ovaries. At E6–6.5 (HH29), gonads begin morphological differentiation into testes in ZZ embryos or ovaries in ZW embryos. Sex-specific gonadal morphology emerges at this time, and genes important for ovary or testis development show sex-biased expression.

Genes involved in gonadal sex differentiation have been analysed extensively in chickens^2,3^. Several key genes involved in mammalian gonadal sex differentiation are conserved in chickens, including genes up-regulated in males (e.g., *doublesex* and *mab-3*-related transcription factor 1 (*DMRT1*), sex-determining region Y-box 9 (*SOX9*), anti-Mullerian hormone (*AMH*) and hemogen (*HEMGN*)), genes upregulated in females (e.g., forkhead box L2 (*FOXL2*) and cytochrome P450 family 19 subfamily A member 1 (*CYP19A1*, also known as aromatase)), and genes upregulated in both sexes (e.g., nuclear receptor subfamily 5 group A member 1 (*NR5A1*, also known as *SF-1*))^4–15^. Gene expression has been evaluated in embryonic gonads from E4.5 (HH24) to E6 (HH29) by RNA-sequencing in the chicken^16,17^. These data indicated that over 1,000 genes are transcribed in a sex-biased manner at E6.5 (HH29), and the majority of these become biased between E4.5 (HH24) and E6 (HH29). Novel genes and pathways that are activated in a sex-specific manner at the time of gonadal sex differentiation have been identified, emphasizing the utility of the RNA-seq approach.

The chicken is a common animal model. A number of transgenic chickens have been generated by retroviral infection^18–21^ and transposon systems^22,23^. For several genes involved in gonadal sex differentiation, over-expression or knockdown experiments have been performed^4,5,8–10,24,25^. Genetic editing tools, such as transcription activator-like effector nuclease (TALEN) and clustered regulatory interspaced short palindromic repeats (CRISPR)/CRISPR-associated protein 9 (CRISPR/Cas9), have been applied to modify the chicken genome. Null mutants have been generated for two egg white genes, ovalbumin and ovomucoid^26,27^, as well as DEAD-box helicase 4 (*DDX4*; also known as vasa), which is essential for the proper formation and maintenance of germ cells^28^. However, these methods are dependent on germline-mediated chimerism, which is time-consuming because individuals in the first generation are always mosaic. In addition, the introduction of mutations in female-specific genes on the W chromosome is nearly impossible because female-derived primordial germ cells are difficult to culture^29,30^.

We propose that the Japanese quail (*Coturnix japonica*) is a useful model for studies of the molecular mechanisms underlying gonadal sex differentiation. The Japanese quail is a small species in the family Phasianidae and is a well-established animal model for biological research. The Japanese quail benefits from its easier handling and shorter generation time than those of other Phasianidae species, such as the chicken. It requires only 16 days to hatch and 6 to 8 weeks of age to reach sexual maturity. In addition, the Japanese quail shows excellent reproductive performance (egg production, fertility and hatchability), comparable or superior to those of the chicken^31^. The Organization for Economic Co-operation and Development (OECD) recommends the use of the Japanese quail as a model for avian safety assessments^32^. Developing embryos of the Japanese quail can be easily cultured ex vivo and manipulated. In particular, it is worth noting that in vitro fertilization^33,34^ and complete culture from the single-cell stage to hatching^35^ have only been demonstrated in the Japanese quail to date. Current in vitro technologies in the species could be powerful tools for the production of genome-edited animals. Thus, the Japanese quail may be an effective alternative to the chicken as a laboratory research animal.

In this study, we performed comprehensive gene expression profiling of embryonic gonads at HH27 (just after sex determination and before morphological differentiation between ZZ and ZW embryonic gonads), HH31 (after morphological differentiation) and HH38 (proceeding differentiation) in the Japanese quail by mRNA-seq. We further classified genes according to the extent of change between stages and performed GO analyses to establish the potential functions of these genes in reproduction. Our results demonstrate that expression profiling using RNA-seq data for embryonic gonads is effective for the identification of novel candidate genes involved in gonadal sex differentiation. Further analyses indicated that the expression levels and localization of key genes for gonadal sex differentiation were consistent with those in chicken embryos, except for *HEMGN*. Based on our findings, the Japanese quail is an effective model for the identification of novel genes involved in gonadal sex differentiation.

## Results

### Gene expression profiling

**Figure 1:**
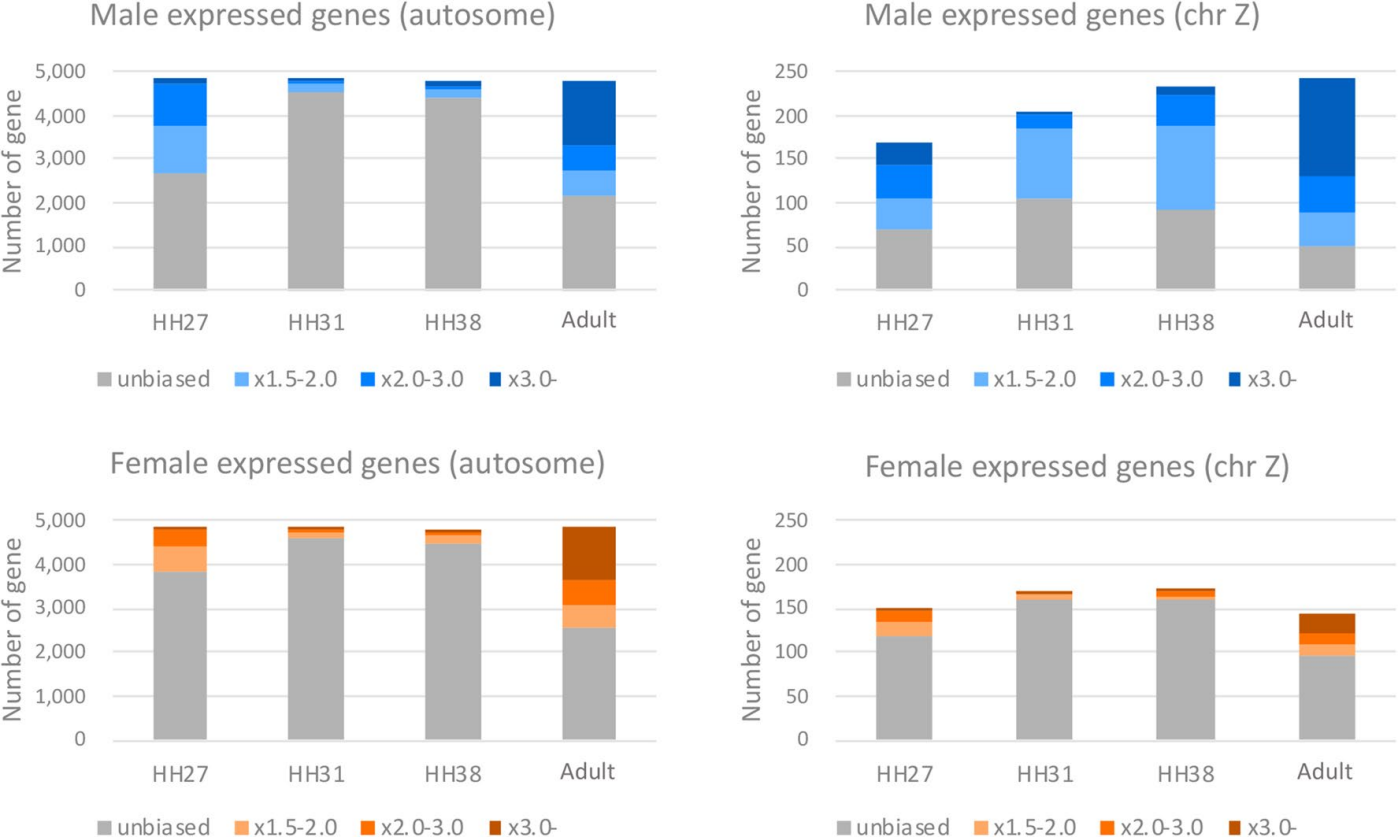
Top 5,000 genes with high FPKM value in male (ZZ) and female (ZW) at the following four stages: embryonic gonads at HH27, HH31, and HH38, and the adult testis or ovary. Bar graphs were showed separately for autosomes (left side) or the Z chromosome (right side). Genes were classified into the following four groups according to fold change between sexes at the same stage: unbiased (grey), × 1.5–2.0 fold change (light blue for male and light orange for female, respectively), × 2.0–3.0 (blue and orange), and × 3.0- (dark blue and brown).

We conducted RNA-seq analysis focusing on changes in the expression level of genes. Total RNAs extracted form gonads of over than five individuals of each stage and sex were used. The RNA-seq data were used to obtain gene expression profiles for Japanese quail gonads at four developmental time points in males and females: HH27 (just after sex determination and before morphological differentiation between ZZ and ZW embryonic gonads), HH31 (after morphological differentiation), HH38 (proceeding differentiation) and adult. An MA-plot of TMM normalized FPKM is shown in Fig. S1. To confirm trends of gene expression patterns at each of the stages and chromosomes, the top 5,000 genes with high FPKM value at each samples are shown in Fig. 1, Fig. S2 and Table S1. We classified the top 5,000 genes into the following four groups according to fold change between sexes at the same stage: unbiased (- × 1.5 fold change), × 1.5–2.0, × 2.0–3.0 and × 3.0- fold change. A proportion of genes with fold-change × 2.0- on the autosomes was confirmed to be higher in the adult testis (43.1%) and ovary (36.6%) than at the other three early developmental time points. We also confirmed that the tendency did not change even if the threshold value of fold-change (× 1.5 or × 3.0) was changed. In males, genes located on the Z chromosome showed a similar pattern to that of autosomal genes, and the proportion of Z chromosomal genes with fold-change × 2.0- was higher than that on the autosomes in all developmental stages. It was also confirmed that the proportion of genes with fold change × 2.0- (× 1.5- and × 3.0, too) on chromosome Z is higher in males than in females at all stages. In particular, genes with fold-change × 2.0- accounted for 63.8% and 23.6% of Z chromosomal genes in adult males and females, respectively, and likewise genes with fold-change × 1.5- accounted for 79.4% and 34.0%, respectively. The top 3,000 and 10,000 genes showed similar features as the top 5,000 genes (Table S2 and S3). We also confirmed that similar results were obtained using GFOLD and isoDE2, which are designed for gene expression analysis using RNA-seq data without biological replicates (Figs. S3 and S4).

**Figure 2:**
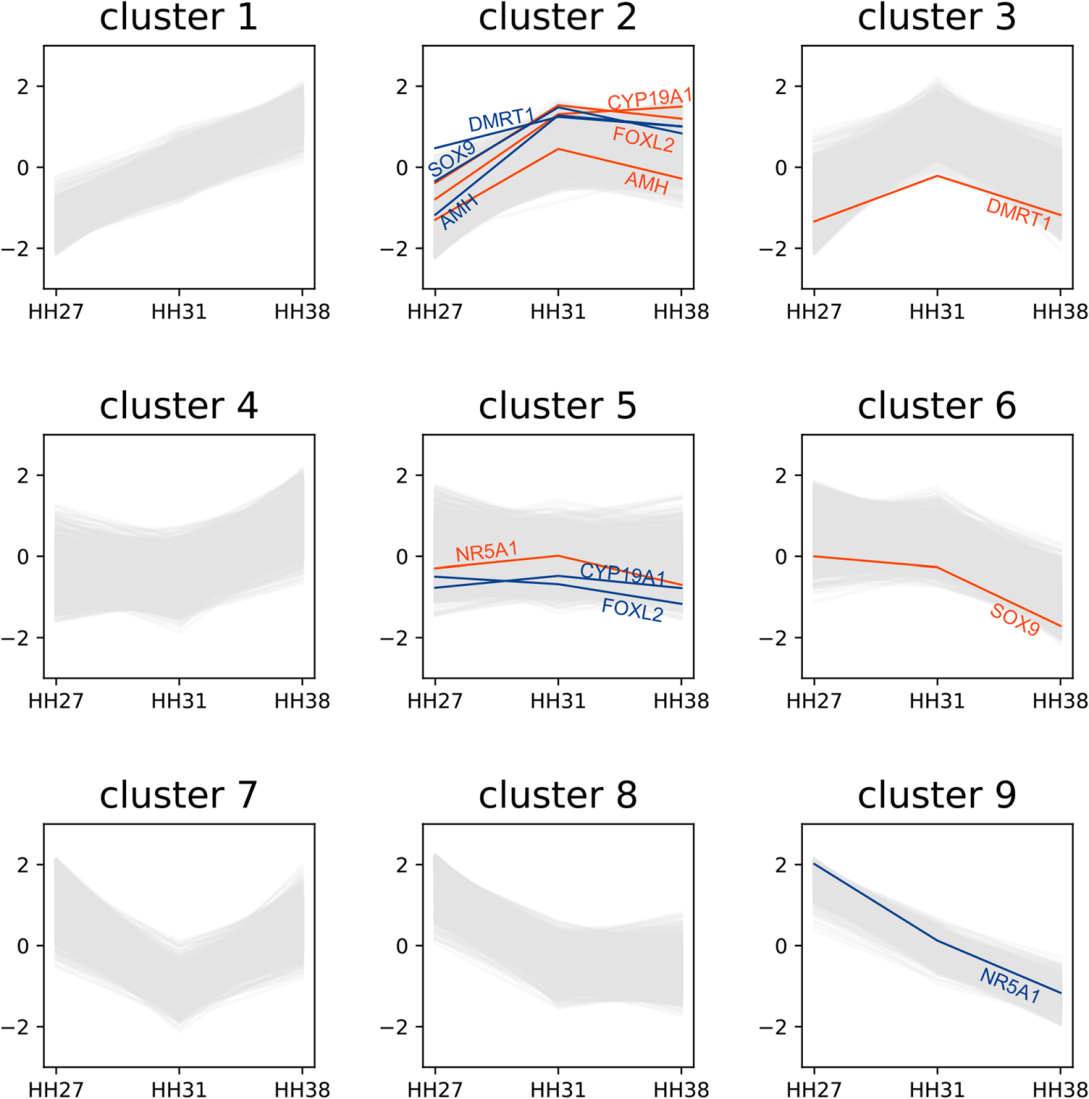
Expression patterns of nine clusters during gonadal sex differentiation. Between HH27 and HH31, gene expression levels increased in clusters 1, 2 and 3, remained constant in clusters 4, 5 and 6, and decreased in clusters 7, 8 and 9. Similarly, between HH31 and HH38, gene expression levels increased in clusters 1, 4 and 7, remained the same in clusters 2, 5 and 8, and decreased in clusters 3, 6 and 9. Blue and orange lines indicate genes related to gonadal differentiation in males (ZZ) and females (ZW), respectively.

Focusing on the 4,815 genes with TMM normalized FPKM values of ≥ 30, we classified expression patterns into nine clusters according to the extent of change in two comparisons, HH27–HH31 and HH31–HH38 (Table 1 and Fig. 2; 3 × 3 clusters). Cluster 2 (up–flat), including 495 and 310 genes expressed in males and females, respectively, contained several genes involved in gonadal sex differentiation, including *DMRT1*, *SOX9* and *AMH* in males (blue lines in cluster 2, Fig. 2), and *FOXL2* and *CYP19A1* in females (orange lines in cluster 2, Fig. 2). These genes displayed a common trend in which the expression level increased during HH27–HH31 and remained constant thereafter. Among the genes classified into cluster 2, those whose fold-change between males and females was ≥ 2 at HH31 comprised 52 genes in males (Table S4) and 40 genes in females (Table S5). *NR5A1,* which activates *CYP19A1* in the chicken ovary, was assigned to cluster 5 (flat–flat) for females. *HEMGN,* involved in testis differentiation in the chicken, was excluded from the clustering analysis owing to its low expression level in the Japanese quail.

**Table 1:** Gene expression pattern in embryonic gonads of Japanese quail.

Cluster	Expression pattern		Number of genes			Genes known to be involved in sex differentiation	
	HH27-31	HH31-38	Male	Female	Total	Male	Female
Cluster 1	↗	↗	320	192	512		
Cluster 2	↗	→	495	310	805	*DMRT1, SOX9, AMH*	*FOXL2, CYP19A1, AMH*
Cluster 3	↗	↘	558	145	703		*DMRT1*
Cluster 4	→	↗	363	974	1,337		
Cluster 5	→	→	139	1,353	1,492	*FOXL2, CYP19A1*	*NR5A1 *
Cluster 6	→	↘	562	1,282	1,844		*SOX9*
Cluster 7	↘	↗	507	178	685		
Cluster 8	↘	→	1,090	256	1,346		
Cluster 9	↘	↘	781	125	906	*NR5A1*	
		Total	4,815	4,815	9,630

### GO analysis

To investigate the functional characteristics of the 92 genes with expression differences between males and females in cluster 2, we performed a GO analysis. In total, 13,243 of the 16,037 protein-coding genes of the Japanese quail were annotated to one or more GO terms. The criteria for significance were FDR < 0.05 and a hierarchical depth of up to three. Focusing on the biological process domain, 92 genes (52 in males and 40 in females) were assessed. GO terms related to developmental processes including reproductive structure development and developmental processes involved in reproduction were significant (Table 2).

**Table 2:** GO terms in the "cluster 2" genes exhibiting different expression levels between males and females.

# GO	Term	Ratio_in_study	Ratio_in_pop	Depth	p_fdr
GO:0,048,608	reproductive structure development	7/92	99/20,132	3	0.000
GO:0,048,856	anatomical structure development	21/92	1279/20,132	2	0.002
GO:0,032,502	developmental process	26/92	2064/20,132	1	0.002
GO:0,003,006	developmental process involved in reproduction	8/92	207/20,132	2	0.008
GO:0,048,869	cellular developmental process	18/92	1229/20,132	2	0.014
GO:0,050,793	regulation of developmental process	17/92	1116/20,132	3	0.014
GO:0,051,239	regulation of multicellular organismal process	18/92	1291/20,132	3	0.020

### Genes with dimorphic expression involved in gonadal sex differentiation

**Figure 3:**
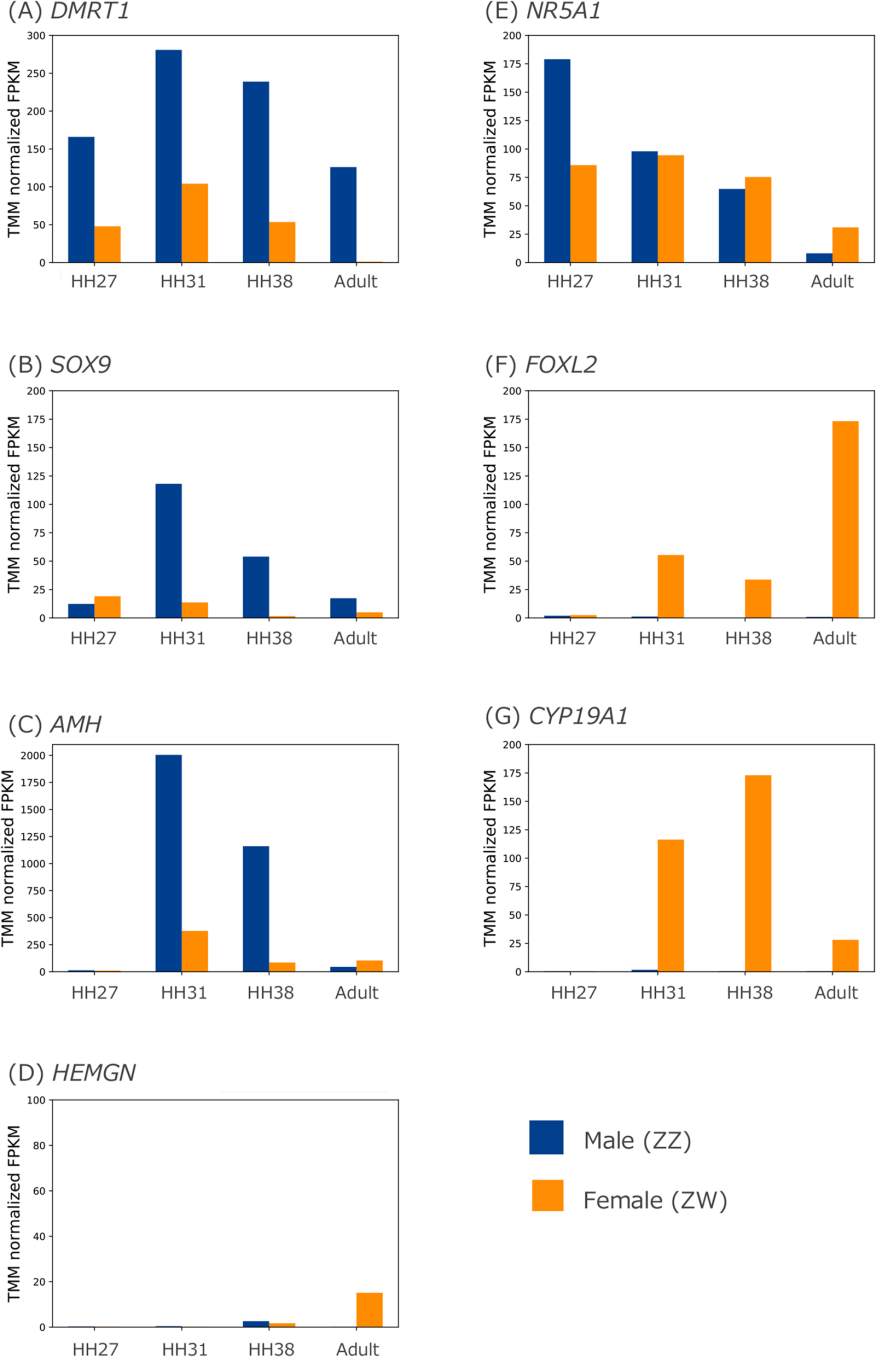
Comparison of expression patterns of seven genes between male (ZZ) and female (ZW) gonads using TMM normalized FPKM values. The expression patterns of *DMRT1* (**A**), *SOX9* (**B**), *AMH* (**C**), *HEMGN* (**D**), *NR5A1* (**E**), *FOXL2* (**F**) and *CYP19A1* (**G**) are shown based on TMM normalized FPKM values in embryonic gonads at HH27, HH31, HH38 and the adult testis or ovary. Blue and orange bars indicate males (ZZ) and females (ZW), respectively.

We analysed the expression patterns of seven genes involved in gonadal sex differentiation, *DMRT1*, *SOX9*, *AMH*, *HEMGN*, *NR5A1*, *FOXL2* and *CYP19A1*, using RNA-seq data. We obtained RNA-seq data from the embryonic gonads of the Japanese quail at HH27, HH31 and HH38 and from the testis and ovary of an adult for comparisons between males (ZZ) and females (ZW). FPKM values normalized using the TMM method for seven genes were evaluated (Fig. 3).

*DMRT1* expression was detected at HH27 and reached a peak at HH31 in males and females (Fig. 3A). Throughout all examined stages, expression levels were higher in males than in females. *DMRT1* expression remained high in adult males, unlike the other six genes, which decreased over time. *SOX9* expression was slightly higher in females than in males at HH27 (Fig. 3B). Beyond HH27, expression increased in males but remained at extremely low levels in females. Obvious expression of *AMH* was not detected at HH27; however, expression increased dramatically in males after HH31 (Fig. 3C). In adults, although the *AMH* expression level was low, it was higher in females than in males. Although *HEMGN* expression was slightly higher in males than in females at HH38, it was quite low at all embryonic stages in both sexes (Fig. 3D). In adults, *HEMGN* expression was only detected in females.

*NR5A1* expression was approximately two-fold higher in males than in females at HH27 (Fig. 3E). *NR5A1* expression was similar in males and females at HH31, since expression in males decreased. In adults, the expression pattern was reversed, with higher levels in females than in males. *FOXL2* and *CYP19A1* showed female-specific expression throughout all examined stages (Fig. 3F and G). A peak of *FOXL2* expression was detected at embryonic stage HH31, and the highest expression among all stages was observed in adult females. *CYP19A1* expression was higher than *FOXL2* expression during embryonic stages, with a peak at HH38. Adult females showed low expression of *CYP19A1*.

**Figure 4:**
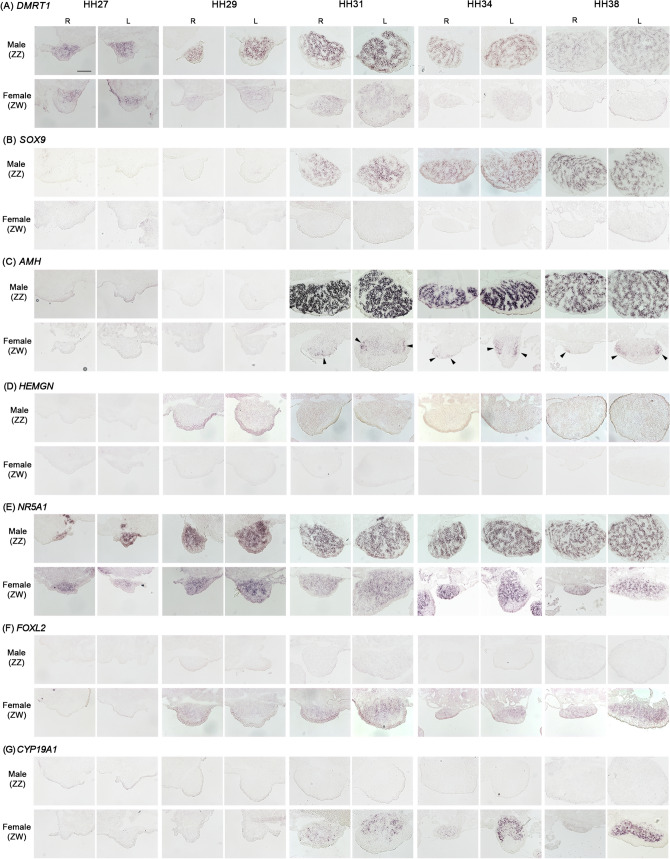
Expression patterns of seven genes in embryonic gonads of the Japanese quail. Expression patterns of *DMRT1* (**A**), *SOX9* (**B**), *AMH* (**C**), *HEMGN* (**D**), *NR5A1* (**E**), *FOXL2* (**F**) and *CYP19A1* (**G**) were examined by in situ hybridization in frozen gonad sections of ZZ (male) and ZW (female) at HH27, HH29, HH31, HH34 and HH38. R and L indicate right and left gonads, respectively. Scale bar indicates 100 µm.

We used in situ hybridization to confirm the localization of these seven genes in Japanese quail embryonic gonads at five developmental stages, HH27, HH29, HH31, HH34 and HH38. *DMRT1* signals were localized to the gonadal medulla of males (ZZ) at all examined stages (Fig. 4A) and in the gonadal medulla of females (ZW) from HH27. However, the signals were weaker in females than in males and almost undetectable after HH34. *SOX9* signals were detected in the gonadal medulla of males from HH31 (Fig. 4B) and were not detected in female gonads at any stages. A similar pattern was observed for *AMH* hybridization in male gonads (Fig. 4C); however, the signals in males were the strongest among the three male-biased genes. Although the signals were weak in female gonads at all stages, expression was detected at HH31, 34 and 38 (arrowheads in Fig. 4C). In *HEMGN*, quite weak signals were observed in males from HH29 to HH38 (Fig. 4D). *DMRT1* showed the earliest expression (from HH27) among all four male-biased genes.

Relatively strong *NR5A*1 signals were observed in the gonadal medulla of males and females at all examined stages (Fig. 4E). Two female-biased genes, *FOXL2* and *CYP19A1*, showed female-specific expression (Fig. 4F and G, respectively). *FOXL2* was detected earlier than *CYP19A1* (i.e., HH29 and HH31, respectively). The signals of both genes were localized to the gonadal medulla of females, but *CYP19A1* expression was stronger than *FOXL2* expression. Overall, the mRNA expression and localization patterns were consistent with the patterns reported in chicken embryos, with the exception of *HEMGN*.

## Discussion

Genes involved in gonadal sex differentiation show sex-biased expression and regulate testicular or ovarian development. Several of these genes have been evaluated in knockout mouse models and in humans with disorders of sex development. However, gonadal sex differentiation is not well-understood and likely involves a number of unknown genes. In birds, the molecular mechanism underlying gonadal sex differentiation has implications for poultry breeding. We found that embryonic gonads at HH27 (just after sex determination) show the highest percentage of genes with sex-biased expression on the autosomes in the three embryonic stages. Many unknown genes are differentially expressed to promote gonadal differentiation after sex determination. The number of Z chromosomal genes showed male-biased expression was higher than that on the autosomes in all stages. We also confirmed that male-biased expression genes distributed throughout the Z chromosome, and was not biased to small regions (Fig. S5). This indicates that Z chromosomal genes are not completely dosage compensated in birds. Previous studies have reported that the mean expression levels of Z-linked genes are 1.6-fold higher in males than in females^36–38^. A similar result has been obtained in an RNA-seq analysis of blastoderms and embryonic gonads of chickens^15^. We could not identify W genes owing to insufficient sequence information for the Japanese quail. The enrichment of genome sequence information for the W chromosome of the species should be a focus of research.

We classified expression patterns into nine clusters according to the extent of change between HH27 and HH31 and between HH31 and HH38. Five genes involved in gonadal sex differentiation, *DMRT1*, *SOX9* and *AMH* in males and *FOXL2* and *CYP19A1* in females, were assigned to cluster 2 (up–flat). These results indicate that genes involved in gonadal sex differentiation tend to increase after sex determination (HH27–HH31) and remain at a constant level during subsequent sex differentiation (HH31–HH38). Genes in cluster 2 were associated with GO terms related to developmental processes, including reproductive structure development, anatomical structure development, developmental process, developmental processes involved in reproduction, cellular developmental process and regulation of developmental process. Our expression profiling approach using RNA-seq data for embryonic gonads was effective for the identification of candidate genes not known to function on gonadal sex differentiation. In particular, the genes classified into cluster 2 (52 and 40 genes in males and females, respectively) (Table S4 and S5) are candidates for further functional assays.

The best candidate master testis-determining gene is Z-linked *DMRT1* in birds lacking mammalian sex determinant *Sry*/*SRY* (sex-determining region Y). The chicken homolog of *DMRT1* is expressed more highly in undifferentiated gonads of males than in females, and a high expression level is maintained in male gonads during testis development. In the Japanese quail, we also detected higher *DMRT1* expression in male embryonic gonads at HH27, which was the earliest expressed male-biased genes, and the expression continued after HH27. These results indicate that *DMRT1* is a conserved gene with key functions in testis determination in the Japanese quail. We also observed male-biased expression of *SOX9* and *AMH* in embryonic gonads after HH31 in the Japanese quail. In placental mammals, SRY directly activates *Sox9*/*SOX9* by binding to the *Sox9*/*SOX9* enhancer together with NR5A1 in the undifferentiated gonads of XY embryos^39^. Subsequently, SOX9 directly regulates *Amh*/*AMH* expression by binding to the *Amh*/*AMH* proximal promoter together with other transcription factors^40–47^. However, in chickens, there is a time-lag between the initial expression of *DMRT1* and *SOX9*, which occur at days 3.5 and 6.5, respectively^48^. Furthermore, *AMH* mRNA is expressed prior to *SOX9* mRNA^7^. Therefore, other factors, probably chicken (birds)-specific factors, must be components of the molecular cascade between *DMRT1* and *SOX9*. We further found that *SOX9* is expressed later than *DMRT1* and *AMH* in the Japanese quail, indicating that the molecular cascade between these genes is conserved in the two species.

By contrast, we did not detect significant *HEMGN* expression in Japanese quail gonads. *HEMGN* is located on the Z chromosome and was first reported as a chicken-specific factor for testis differentiation by mediating *SOX9* regulation under *DMRT1*^10^. In mice, *Hemgn* (also known as *EDAG* in humans) is a hematopoietic tissue-specific gene encoding a nuclear protein^49^. Although the gene is not expressed in the gonads during embryogenesis in mammals, *HEMGN* is expressed in hematopoietic tissues and in early embryonic gonads of male chickens^10^. In ZW embryonic gonads masculinized by aromatase inhibitor treatment, *HEMGN* expression is induced. ZW embryos overexpressing *HEMGN* have masculinized gonads with increases in the male marker genes *DMRT1* and *SOX9* and decreases in the female marker genes aromatase and *FOXL2*. Furthermore, the distribution of germ cells shows a testis-like pattern. These findings suggest that *HEMGN* is specifically involved in gonadal differentiation in the chicken. However, *HEMGN* mRNA expression has not been detected in embryonic gonads of the emu and zebra finch^50^. We confirmed a lack of detectable *HEMGN* expression in the Japanese quail, belonging to the order Galliformes, along with the chicken, although the identity of mRNA sequences of *HEMGN* is 86.24% between chicken (XM_430508) and Japanese quail (XM_032441476). Accordingly, the role of this gene in gonadal sex differentiation could be limited to the chicken and closely related species.

*FOXL2* is a conserved gene involved in ovary development in vertebrates^13–15^. The expression patterns of *FOXL2* and *CYP19A1* are highly correlated in the developing ovary in chickens^12^. *CYP19A1* is also important for ovary development because it encodes an enzyme (aromatase) responsible for converting androgens to estradiol. *FOXL2* is expressed just prior to *CYP19A1*, suggesting that it directly or indirectly regulates aromatase transcription in chickens. We observed nearly identical expression patterns in the Japanese quail. *FOXL2* was initially detected at HH29, before the expression of *CYP19A1*. These results indicate that the functions of two female-biased genes in ovary development are conserved in the Japanese quail. All examined genes other than *HEMGN* showed similar expression and localization patterns in embryonic gonads to those in the chicken, suggesting functional conservation. Our results confirm that these genes can be used as molecular markers for studies of gonadal sex differentiation in the Japanese quail. More broadly, our gene profiling and expression analyses are expected to contribute to the identification of novel candidate genes involved in gonadal sex differentiation in birds.

## Materials and methods

### Animals and ethics statement

Fertilized Japanese quail eggs were purchased from Motoki Corporation (Saitama, Japan). Fertilized eggs were incubated at 37.5 °C. The sex of each embryo was determined by PCR genotyping using genomic DNA as the template^51^. The developmental stage of each embryo was determined based on a previous report by Ainsworth et al.^52^.

All animal experiments described in this study were approved by the Institutional Animal Care and Use Committee of National University Corporation Hokkaido University and were performed in accordance with the Guidelines for the Care and Use of Laboratory Animals issued by Hokkaido University. This study did not involve any human participants or specimens.

### RNA extraction and cDNA synthesis

Total RNA was extracted from embryonic gonads of the Japanese quail at HH27, HH31, HH38, and adult testis and ovary using an RNeasy Kit (Qiagen, Hilden, Germany) according to the manufacturer’s instructions. We used left gonads of embryo and left testes and ovaries of adult, because Japanese quail shows asymmetric development of gonads that right gonads are gradually depress and fail to develop in ZW female. RNA was treated with DNase I and reverse-transcribed using SuperScript III reverse transcriptase (Thermo Fisher Scientific, Waltham, MA, USA) and an oligo(dT) primer.

### RNA-seq

Total RNA was quantified using a Bioanalyzer and RNA 6000 Nano Kit (Agilent, Santa Clara, CA, USA). Total RNA with an RIN value exceeding 9.7 was used for subsequent experiments. Using 200 ng of total RNA as an input, the libraries for RNA-seq were prepared using the SureSelect Strand-Specific RNA Library Preparation Kit (Agilent) according to the manufacturer’s protocol. Then, 100-bp paired-end reads were obtained using the HiSeq3000 platform (Illumina, San Diego, CA, USA). Sequencing data have been deposited in the DDBJ Sequence Read Archive under accession numbers DRA007208 and DRA008738.

### Gene expression profiling

Total RNAs were extracted from gonads of over than five individuals of each stage and sex. We conducted RNA-seq analysis using the extracted RNAs focusing on changes in the expression level of genes. Firstly, for the following analyses, low-quality and adaptor sequences in RNA-seq reads were removed using Platanus_trim v1.0.7 (https://platanus.bio.titech.ac.jp/pltanus_trim). Fragments per kilobase of transcript per million mapped reads (FPKM) were estimated using RSEM (1.3.0)^53^. We used RefSeq Japanese quail genome (*Coturnix japonica* 2.1, GCF_001577835.1) eliminating a mitochondrial sequence as a reference genome. In RSEM, RNA-seq reads were mapped to the reference genome using bowtie2-2.3.4 (–bowtie2-mismatch-rate 0.03)^54^. Next, to enable make comparisons of FPKM values between samples, we applied the trimmed mean of M-values (TMM) normalization method for FPKM values. The TMM normalized FPKM values were calculated using TCC (3.9)^55^. To confirm gene expression patterns at each of the stages or at autosome and chromosome Z, we classified top 5,000 genes with high FPKM value into the following four groups according to fold change between sexes at the same stage: unbiased (- × 1.5 fold change) and × 1.5–2.0, × 2.0–3.0 and × 3.0- fold change. To confirm statistically supported gene expression and fold changes between sexes, we also ran two existing tools, GFOLD v1.1.4^56^ and isoDE2 v1.1.5^57^ with default parameters. The two tools were designed for gene expression analysis using RNA-seq data without biological replicates.

Genes were classified according to their expression patterns at three stages (HH27, HH31 and HH38). The target genes for classification had ≥ 30 TMM normalized FPKM in at least one of six samples (males and females at three stages). The TMM normalized FPKM values were pre-processed according to the common practice of applying quantile normalization, taking the logarithm of data values and converting z-scores. To confirm differences of expression pattern between males and females, we normalized FPKM values of males and at once females for each gene. Based on the difference in the normalized values between HH27 and HH31, the genes in each sex were divided into three by k-means clustering using Euclidean distance (conducted by sklearn library v0.19.2, Python v3.7.0). By this clustering, the differences between the two stages were classified into the following three patterns: increase (up), no change (flat) and decrease (down). Similarly, classification was also performed between HH27 and HH38, and the genes were finally classified into nine groups according to the combination of the differences between HH27 and HH31 and between HH31 and HH38.

### Gene ontology (GO) analysis

To obtain GO terms associated with Japanese quail genes, GO terms for homologous RefSeq chicken genes (GCF_000002315.4) were obtained. GO terms predicted using InterProScan (5.33–72.0)^58^ for Japanese quail protein sequences were merged with the previous GO terms obtained for chicken genes. Next, a GO enrichment analysis was conducted using goatools (0.9.5)^59^. Enriched GO terms with a false discovery rate (FDR) of < 0.05 and hierarchical depth of up to three in a sub-ontology, biological process (BP), were obtained.

### In situ* hybridization*

A fragment of each gene was amplified by RT-PCR using cDNA obtained from gonads as the template. The sequences of primers are listed in Table S6. RNA extraction and cDNA synthesis were performed as described earlier. The CDS sequence of chicken *NR5A1* was inserted in a pBluescript SK. The PCR products of other genes were subcloned using the pGEM T-Easy Vector System (Promega, Madison, WI, USA). cDNA clones were labelled using Digoxigenin RNA Labeling Mix (Roche, Basel, Switzerland) and T7, SP6 or T3 RNA polymerase (MAXIscript; Thermo Fisher Scientific). Hybridization to serial frozen sections was performed as described previously^23^. The incubation temperature was modified to 65–70 °C. Images were captured using a cooled CCD camera (DS-Ri1; Nikon, Tokyo, Japan) mounted on a Nikon ECLIPSE E800 microscope and were analysed using NIS ELEMENTS (Nikon).

## Supplementary information

Supplementary Information.

Supplementary figure S1.

Supplementary figure S2.

Supplementary figure S3.

Supplementary figure S4.

Supplementary figure S5.

Supplementary tables.
