# Supplementary material for: Expression profiling of sexually dimorphic genes in the Japanese quail, Coturnix japonica
Source: Sci Rep. 2020 Nov 30;10:20073. doi: 10.1038/s41598-020-77094-y (PMC7705726; doi:10.1038/s41598-020-77094-y)
Supplement: Supplementary file 1 — Supplementary Information. [file 41598_2020_77094_MOESM1_ESM.docx]

**Expression profiling of sexually dimorphic genes in the Japanese quail, *Coturnix japonica***

Miki Okuno^1, †^, Shuntaro Miyamoto^2, †^, Takehiko Itoh^1^, Masahide Seki^3^, Yutaka Suzuki^3^, Shusei Mizushima^2, 4^, Asato Kuroiwa^2, 4,^ *

^1^ School of Life Science and Technology, Tokyo Institute of Technology, 2-12-1 Ookayama, Meguro-ku, Tokyo 152-8550, Japan

^2^ Biosystems Science Course, Graduate School of Life Science, Hokkaido University, Kita 10 Nishi 8, Kita-ku, Sapporo, Hokkaido 060-0810, Japan

^3^ Department of Computational Biology and Medical Sciences, the University of Tokyo, 5-1-5 Kashiwanoha, Kashiwa 277-8562, Japan

^4^ Division of Reproductive and Developmental Biology, Department of Biological Sciences, Faculty of Science, Hokkaido University, Kita 10 Nishi 8, Kita-ku, Sapporo, Hokkaido 060-0810, Japan

† These authors contributed equally to this work.

*Correspondence should be addressed to A. Kuroiwa, Kita 10, Nishi 8, Kita-ku, Sapporo, Hokkaido 060-0810, Japan, E-mail: asatok@sci.hokudai.ac.jp, Tel/Fax: +81-11-706-2752
