# Supplementary figures and images for: Expression profiling of sexually dimorphic genes in the Japanese quail, Coturnix japonica
Source: Sci Rep. 2020 Nov 30;10:20073. doi: 10.1038/s41598-020-77094-y (PMC7705726; doi:10.1038/s41598-020-77094-y)

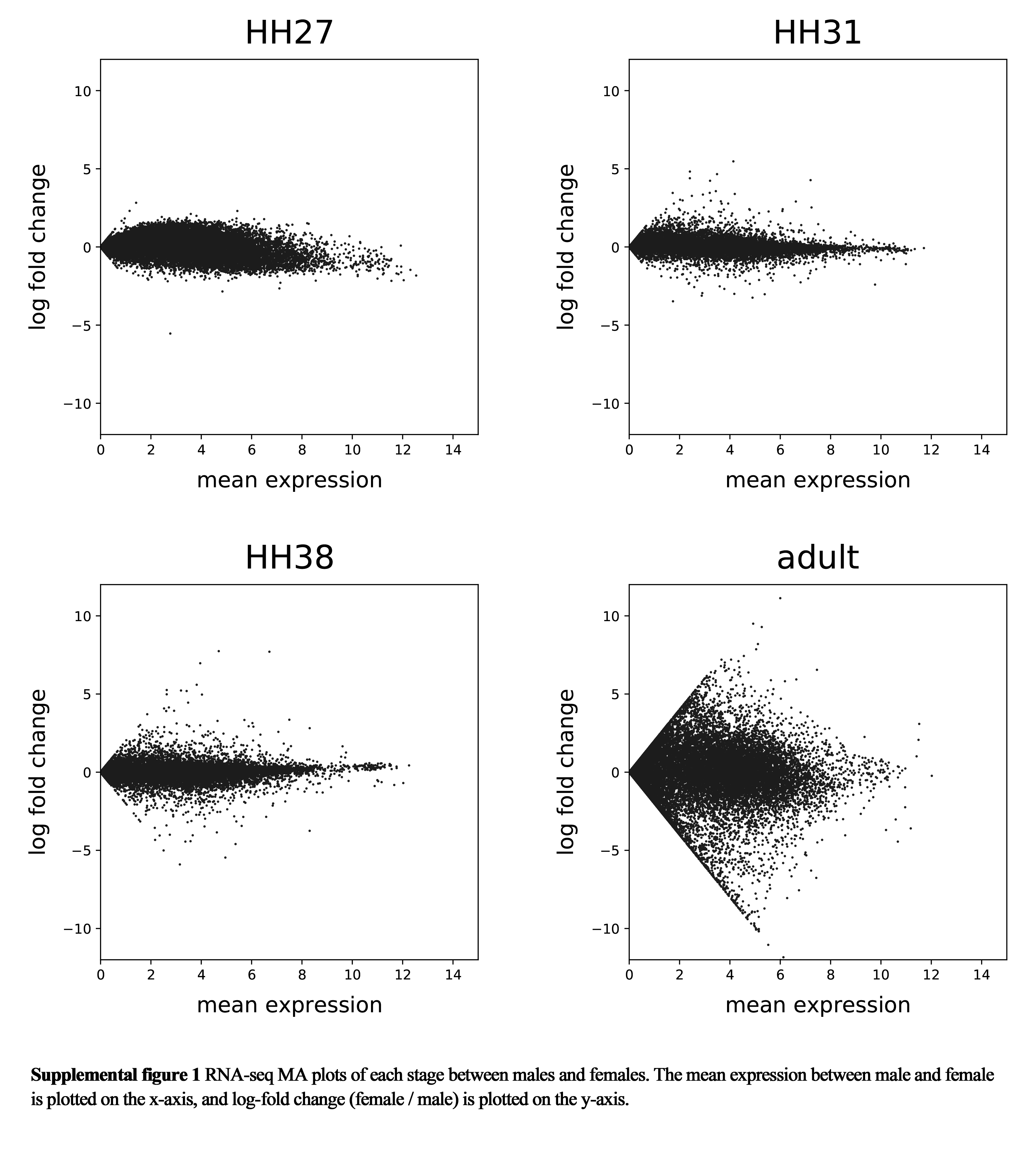

Supplement: Supplementary file 2 — Supplementary figure S1. [file 41598_2020_77094_MOESM2_ESM.tif]

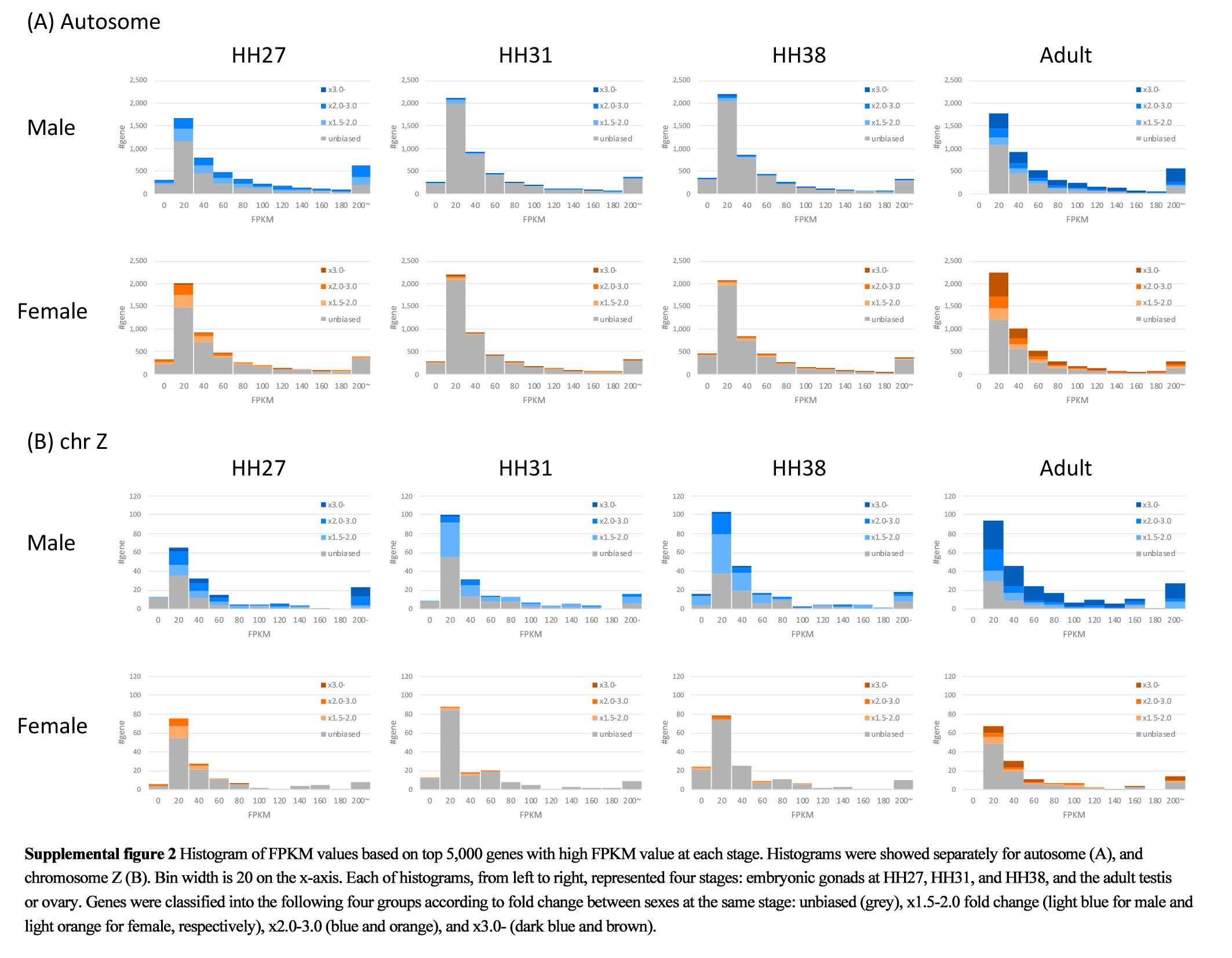

Supplement: Supplementary file 3 — Supplementary figure S2. [file 41598_2020_77094_MOESM3_ESM.tif]

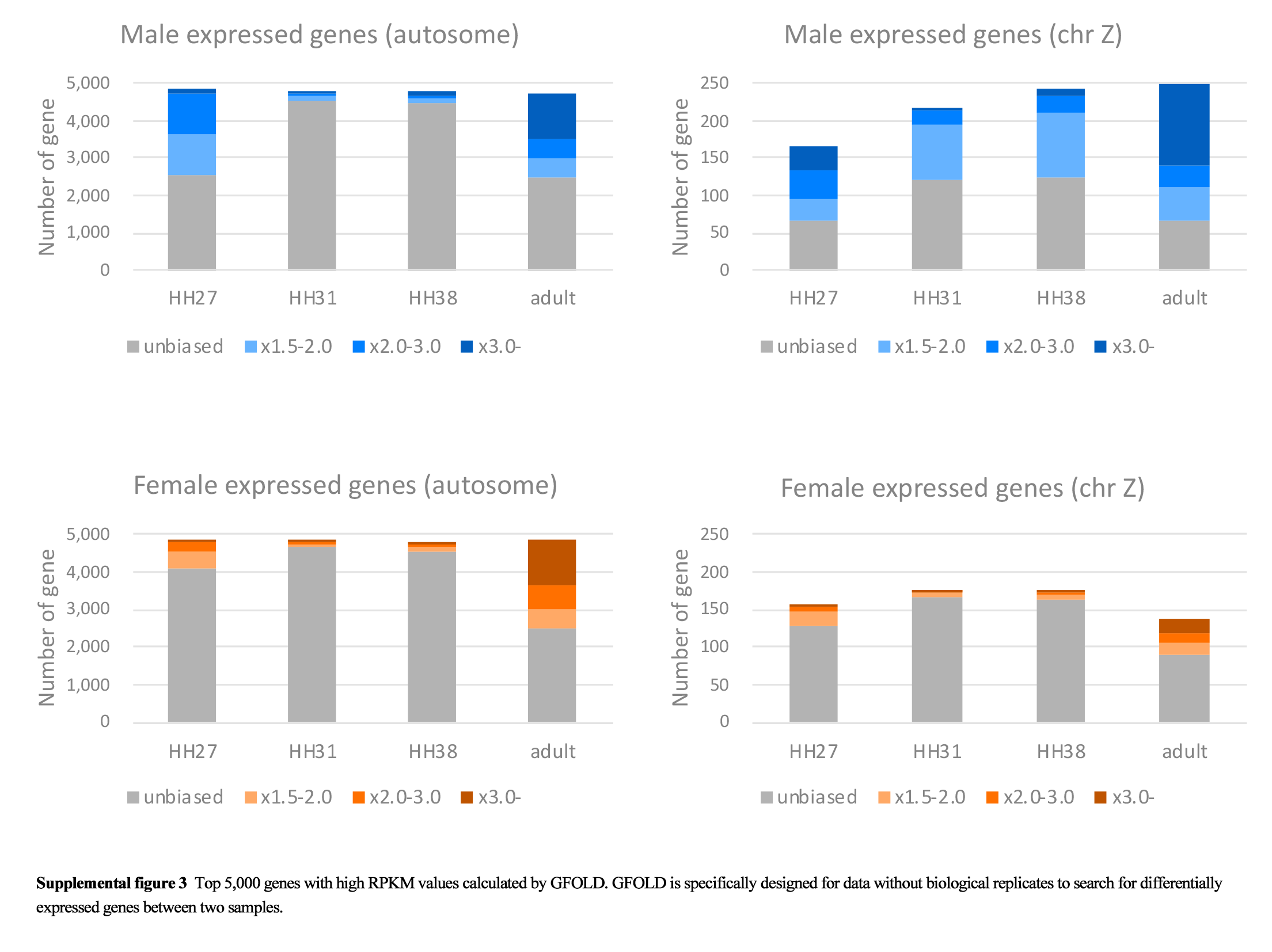

Supplement: Supplementary file 4 — Supplementary figure S3. [file 41598_2020_77094_MOESM4_ESM.tif]

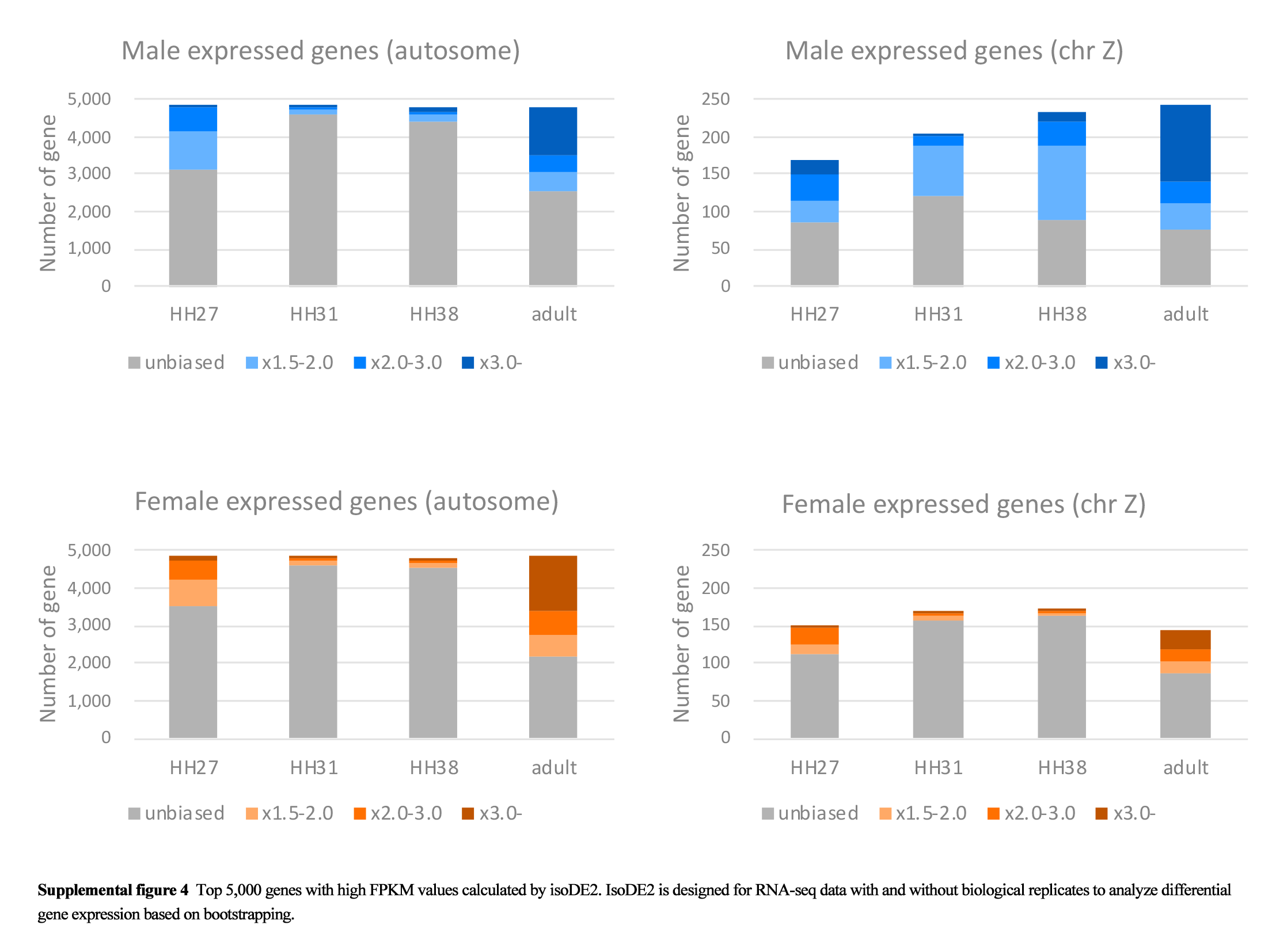

Supplement: Supplementary file 5 — Supplementary figure S4. [file 41598_2020_77094_MOESM5_ESM.tif]

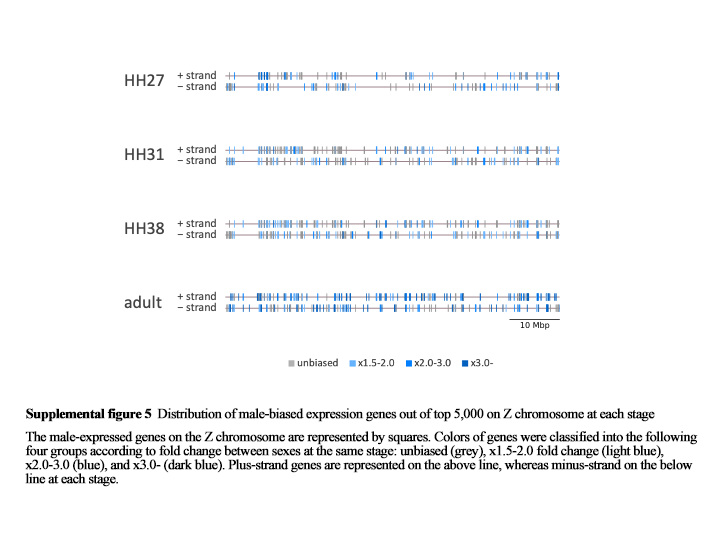

Supplement: Supplementary file 6 — Supplementary figure S5. [file 41598_2020_77094_MOESM6_ESM.tif]
